# Understanding the Inner Workings of Large Language Models in Medicine

**DOI:** 10.12688/f1000research.178855.3

**Published:** 2026-11-13

**Authors:** Georg Fuellen, Hans Jarchow, Johann-Christian Põder

**Affiliations:** 1 Institute for Biostatistics and Informatics in Medicine and Ageing Research, Rostock University Medical Center, Rostock, Germany; 2 Faculty of Theology, University of Rostock, Rostock, Germany

**Keywords:** Natural Language Processing, Large Language Models, Medical Informatics, Ethics, Medical, Clinical Decision-Making

## Abstract

**Background:**

Large language models (LLMs) are increasingly influencing medical practice, education, and research. Their responsible integration into healthcare requires expertise in medical, ethical, practical, and theoretical domains.

**Methods:**

We prompted GPT-o1 to generate examples illustrating how understanding transformer architecture can facilitate output interpretation. Key topics were extracted from its responses, and illustrative cases were identified and cross-checked against the peer-reviewed literature using Consensus.app, an AI-assisted literature-search tool.

**Results:**

Five key topics were identified: (1) anticipating contextual focus in medical reasoning, (2) explaining “generic” or “textbook” responses, (3) understanding strengths and weaknesses in differential diagnosis, (4) explaining ambiguous or contradictory responses, and (5) identifying hallucinations in unfamiliar scenarios. Case examples highlight both benefits and limitations, including accurate attention to salient clinical details, reliance on generalized patterns, risks of base rate neglect in differential diagnosis, challenges of ambiguous prompts, and hallucinations in rare or underrepresented cases.

**Conclusions:**

A theoretical understanding of LLMs is crucial for responsible clinical integration. Distinguishing between well-represented (short head) and underrepresented (long tail) knowledge, recognizing generic responses, and identifying hallucinations are essential competencies. Coupled with medical and ethical expertise, these skills will enable healthcare professionals to leverage LLMs effectively while mitigating risks.

## Introduction

***Background:*** The transformative potential of generative AI and, specifically, of large language models (LLMs) is reshaping contemporary medical theory, practice and education. ^2– 5^ It profoundly influences the future of healthcare as a system, and of its stakeholders. Alongside recognizing the strengths of AI, important questions about its accuracy, and about ethical implications in real-world clinical use have emerged. ^6^ The medical profession must shape its adoption, despite substantial uncertainties about future opportunities and risks. ^7– 9^

As LLM-generated content continues to improve, there is a growing risk that decreasing error rates could make us less cautious in vetting its outputs. This problematic development (“automation bias”) can be further reinforced by evidence that, on certain benchmark tasks, unaided LLMs can match or even exceed the accuracy of human–AI teams. ^10– 15^ We stress that this is a narrow, task-specific performance observation and not a normative claim about clinical autonomy: in healthcare, LLM outputs must remain under human supervision, because the model has no human-like understanding of the tasks it performs or of their consequences, and the clinician remains the responsible sense-maker and decision-maker. To harness the advantages of LLMs sensibly, ensuring a responsible integration of AI in healthcare, we must strive for profound understanding of these technologies, and it is thus crucial to develop and maintain expertise and skills in four key areas ^16– 20^:

1. Medical expertise: To be able to critically evaluate the validity of LLM-generated output.
2. Ethics expertise: To identify potential risks and to address instances of ethical dilemmas and violations of medical-ethical norms.
3. Practical knowledge: Familiarity with LLMs, by informed and critical use.
4. Theoretical knowledge: Knowledge of how LLMs operate, which allows for a more nuanced evaluation of LLM-generated content.

***Our contribution:*** The focus of this paper is on the fourth skill, understanding the inner workings of LLMs to better interpret the contents they generate. One example for this skill is to discern whether the generated content reflects commonly available and well-represented information in their training data (short head knowledge), or whether it refers to underrepresented knowledge (long tail knowledge), potentially causing erroneous responses. These issues are at best mentioned in passing in recent reviews. ^21– 23^ Despite growing interest in interpretability and transparency, there is limited work exploring how a theoretical understanding of LLM mechanisms might enhance clinicians’ ability to evaluate model outputs. Instead of testing clinicians directly, this study uses a self-referential, hypothesis-generating design to identify and illustrate concrete situations in which such theoretical understanding is likely to be interpretively useful, thereby motivating the empirical, systematic studies that would be needed to demonstrate its benefits. Using a self-referential design, we prompted OpenAI’s GPT-o1 to generate examples illustrating how theoretical insights into transformer architecture can facilitate interpretation of medical outputs. Key topics were extracted from its responses and cross-checked against the peer-reviewed literature using Consensus.app, an AI-assisted literature search platform; importantly, this step served to locate corroborating or illustrative studies, not to confirm the validity of the model-generated topics. By examining these examples, we aim to clarify specific situations in which theoretical knowledge of LLMs provides tangible benefits for responsible and informed clinical use.

## Objectives

Overall, we aim to explore how theoretical knowledge of LLMs, specifically their architecture and internal mechanisms, can support the interpretation of model-generated content in medical contexts. In addition, we delineate concrete examples and situational characteristics in which such theoretical understanding provides the most interpretive benefit. We do not aim to measure clinician performance or to establish empirically that theoretical knowledge improves output evaluation; rather, we aim to generate and illustrate hypotheses that future user-based studies can test.

## Methods

### Overview

We conducted an exploratory analysis using AI-assisted tools in a form of model “introspection”. This term is used here as a procedural shorthand for prompting the model to generate explanations of its own outputs: an LLM does not have direct access to its own internal computations, and its explanations of its outputs should not be treated as genuine introspective reports. Mechanistic-interpretability work has shown that the procedure a model reports can diverge substantially from the computation it actually performs. ^24^ We therefore treat the model’s outputs throughout as generated hypotheses to be examined critically by the human authors. An LLM was employed to generate key examples (key topics) that illustrate how theoretical knowledge of transformer architectures can support the interpretation and evaluation of AI-generated outputs in medical contexts. These key topics were then examined and substantiated using an AI-assisted literature search tool.

### Large Language Models (LLMs)

LLMs are neural-network-based text processing tools composed of multi-head self-attention layers and multi-layer perceptrons (MLPs), trained on large volumes of text to predict the next word. During processing, multi-head attention allows the model to maintain several parallel foci on specific parts of the input, potentially connecting dispersed fragments of text, while the MLPs further integrate and transform these representations. ^25, 26^ After a pipeline of attention and MLP layers, the next token is predicted. Throughout this paper, terms such as “attention”, “focus”, “knowledge”, “reasoning”, and “meta-knowledge” are used only in a careful and qualified sense. They do not attribute human-like mental states, beliefs, intentionality, or clinical responsibility to the model. Natural and artificial intelligence must be distinguished, because the mechanisms of LLMs differ from those of human cognition even where their outputs resemble human reasoning. ^27, 28^ Here, we refer to the functional role of statistically generated outputs within distributed, hybrid, and human-interpreted epistemic practices. We avoid attributing literal “understanding” to LLMs: the model does not possess understanding or causal models of disease in the human sense; the regularities it extracts by statistical inference cannot be equivalent to executing evidence-based medicine or employing causal theories of disease. As of mid-December 2024, several LLMs were available; OpenAI’s GPT-o1 (professional mode) was chosen for this study because of its strong generative capabilities. Since this model lacked built-in web search features, its references were often hallucinated, which made an independent validation step necessary.

### Prompting of the generative AI tools

GPT-o1 in professional mode served as the primary tool for generating the key topics related to interpretability and clinical use. Consensus.app, an AI-assisted literature search engine, was used to retrieve peer-reviewed studies relevant to the model-generated key topics and to mitigate the risk of hallucinated citations. All prompts, GPT-o1 outputs, and Consensus.app search results are available in Supplementary Sections A and B.

### Processing of the generative AI output

The exploratory analysis was conducted in three phases. First, we prompted GPT-o1 to propose key topics illustrating how theoretical insights into transformer architectures can support the interpretation of AI-generated text in medical scenarios and applications. We reviewed the output and extracted the relevant topics. To cross-check the outputs and to replace potentially hallucinated references with verifiable ones, we performed a literature search using Consensus.app, incorporating the GPT-o1–generated key topics into the search prompts. From the retrieved results we selected two studies – one focusing on rehabilitation medicine (Zhang et al.) ^29^ and another on clinical interview analysis using synthetic data (Wu et al.), ^30^ – as especially illustrative; this selection was non-systematic and intended to exemplify, rather than to comprehensively or quantitatively test, the key topics. Finally, we compared the model-generated examples with the findings curated through Consensus.app and consolidated them into the final set of five key topics, presented in the Results section. All steps were documented to ensure transparency throughout the exploratory process.

### Ethical considerations

This article does not involve research with human participants or animals. No ethical approval was required.

## Results

Based on the initial prompt, GPT-o1 suggested the following key topics for which it provided example descriptions:

1. Anticipating contextual focus in medical reasoning.
2. Explaining “generic” or “textbook” responses.
3. Understanding strengths and weaknesses in differential diagnosis.
4. Explaining ambiguous or contradictory responses.
5. Identifying hallucinations in unfamiliar scenarios.

Based on the follow-up prompt, Consensus.app suggested examples for the key topics, and we thus investigated the work of Zhang et al. to address key topics (1), (2), and (5) (see Table 1). In their study, the authors employ a stroke case to evaluate the capability of LLMs in generating rehabilitation recommendations and ICF codes. We also investigated Wu et al., who describe how LLMs can facilitate clinical interview analysis through their “CALLM” framework, a method for AI-driven synthetic data augmentation. This investigation contributed to the analysis of key topics (3) and (4) (see Table 2).

**Table 1.** Key Topics (1), (2) and (5), described by GPT-o1, exemplified by Consensus.app.

GPT-o1 explanation for the key topic	Consensus.app explanation for the example	Quotes from the paper (Zhang et al.)	Synopsis
**(1) Anticipating contextual focus in medical reasoning**			
“Transformers use attention mechanisms to “focus” on specific parts of the input sequence. In a medical dialogue, the model might concentrate heavily on a particular symptom, lab value, or part of a patient’s history. Understanding this can help clinicians and medical researchers appreciate why a model’s conclusion might hinge on early-mentioned or highly salient details rather than a broad synthesis of all information.”	“Understanding the architecture of transformer models, particularly the attention mechanism, can help anticipate how these models focus on specific parts of the input text when generating medical reasoning. For instance, in the context of rehabilitation medicine, ChatGPT-4 was able to generate comprehensive medical and rehabilitation prescriptions by focusing on relevant parts of the input case description, demonstrating its ability to prioritize contextually important information.”	“[the LLM] also demonstrated the ability to propose specified approaches for each rehabilitation therapy.”	LLMs can indeed prioritize specific characteristics of a case or patient, and complex scenarios can be addressed through the parallel operations of multiple attention heads. While this capability offers advantages in focusing on critical aspects, it may also have drawbacks, particularly when LLMs fail to correctly integrate and synthesize the resulting information.
**(2) Explaining “generic” or “textbook” responses**			
“MLP layers in transformers combine information gathered by attention heads into higher-level abstractions. When operating on medical queries, these layers may rely on well-learned, “standardized” patterns from training data (e.g., common guidelines or textbook phrasing) instead of tailoring responses to unusual clinical nuances. Understanding the MLP’s integrating role explains why a model might revert to a generic standard-of-care response even when presented with a complex or unique patient scenario.”	“In the study on rehabilitation medicine, ChatGPT-4 produced broader and more general prescriptions that were consistent with textbook answers, indicating its reliance on learned generic medical knowledge.”	“Compared with standard answers, the large language model generated broader and more general prescriptions in terms of medical problems and management plans, rehabilitation problems and management plans, as well as rehabilitation goals.”	Referring to knowledge that is well-represented in the training data (short head knowledge) can result in “generic” or “textbook” responses, raising concerns, however, about their adequacy when addressing atypical cases and patients.
**(5) Identifying hallucinations in unfamiliar scenarios**			
“Transformers are trained on patterns within a certain data distribution. When confronted with rare conditions, novel treatments, or unusual clinical contexts, the model’s learned patterns may not apply. Attention could be misdirected, and the MLP layers might produce “hallucinated” content because they have no solid internal representation for the out-of-distribution input.”	“[…] while ChatGPT-4 made an error in the ICF category, it accurately generated ICF codes, highlighting the model’s potential to hallucinate in less familiar contexts.”	“A thorough review of the standard clinical ICF code assigned by 2 PMR clinicians was then conducted, comparing it with the table produced by the GPT-4 model (Table II). The 3-digit codes generated by the LLM were accurate (…) However, an error was found when reviewing the case record in the body structures category (s730). The patient had had a stroke, and the original impairment should have been classified as affecting the right precentral gyrus (s110.1), as outlined in the case section. Instead, the table displayed the damage as being in “the upper extremity, left hand.”“	LLM responses may exhibit hallucinations when referring to “long tail” knowledge that is not well-represented in the training data. This is hypothesized to be the case for the “body structures category”. *Then again, the LLM-generated explanations in this table are not necessarily correct either.* A simpler hypothesis regarding the LLM failure is that it did not know or did not consider that the “body structures category” is supposed to refer to the primary site of damage (the brain), not to the secondary site (the hand). Any lack of knowledge regarding the reporting of ICF categories may thus be attributed to insufficient training data regarding this meta-level information.

**Table 2.** Key Topics (3) and (4), described by GPT-o1, exemplified by Consensus.app.

GPT-o1 explanation for the key topic	Consensus.app explanation for the example	Quotes from the paper (Wu et al.)	Synopsis
**(3) Understanding strengths and weaknesses in differential diagnosis**			
“Attention layers help identify connections between symptoms and conditions, while MLP layers synthesize these into coherent outputs. Knowing this pipeline is useful when the model suggests a differential diagnosis. If the model posits an unusual condition, it might be because it latched onto a distinctive symptom that strongly correlated with that condition in its training data—even if that condition is clinically improbable.”	“The strengths of transformer models in differential diagnosis can be attributed to their ability to synthesize information from diverse sources, while weaknesses may arise from their lack of real-world clinical experience. The CALLM framework, for example, enhances clinical interview analysis by generating synthetic data that can improve diagnostic accuracy, showcasing the model’s adaptability in learning from augmented datasets.”	“In automated mental health diagnosis, the scarcity and imbalance of clinical data pose considerable challenges for researchers, limiting the effectiveness of machine learning algorithms. To cope with this issue, this paper aims to introduce a novel clinical transcript data augmentation framework by leveraging large language models (CALLM). The framework follows a “patient-doctor role-playing” intuition to generate realistic synthetic data.”	A hypothesis about how LLMs handle differential diagnoses is that multi-head attention may be responsible for the matching of patient data to the sets of symptoms known for disease conditions, but this matching may ignore disease prevalence. Synthetic data may mitigate this weakness because researchers can generate examples following a data distribution they have under control, and provide these examples to the LLM.
**(4) Explaining ambiguous or contradictory responses**			
“When the patient’s presentation is ambiguous or the prompts contain conflicting information, attention mechanisms may distribute focus across multiple, equally plausible interpretations. The MLP layers may fail to resolve these into a single, authoritative answer. Understanding this helps users interpret uncertain or oscillating responses as a reflection of the model’s internal struggle with ambiguity rather than mere randomness.”	“Ambiguities or contradictions in model outputs can often be traced back to the model’s training data or the inherent complexity of medical language. The CALLM framework’s use of a “Response-Reason” prompt engineering paradigm aims to generate diagnostically valuable transcripts, which can help mitigate such issues by providing clearer reasoning paths in the model’s responses.”	“Our “Response-Reason” prompting approach guides LLMs in generating highly authentic clinical interview transcripts for mental disorder diagnosis. This augmentation is tailored to enhance the training dataset, facilitating both FSL [Few-Shot-Learning] and, in certain cases, ZSL [Zero-Shot-Learning].“”This technique […] encouraged it to elucidate the rationale behind the responses, mirroring the profile and characteristics of a simulated patient.”	Contradictory responses can be attributed to ambiguous input, ambiguity within the training data, or ambiguity in the representation of knowledge by the trained model. Specialized prompting techniques may request that the reasoning path of the LLM is made more transparent, enhancing its reasoning capabilities along the way.

### Key topic 1: Anticipating contextual focus in medical reasoning

Key topic (1) illustrates how the LLM’s attention mechanism enables it to identify and prioritize relevant information within a text, connecting specific parts of the text that may be far away in the input stream. In fact, this “focus on specific parts” may be done multiple times in parallel by multiple attention heads and integrated across layers until the final output is generated. The attention mechanism thus allows the model to focus on critical elements – such as specific patient symptoms or lab values – while ignoring less pertinent data. GPT-o1 underscores that clinicians and medical researchers can benefit from understanding that model attention might attend to highly salient details at the expense of a broader synthesis. This focused approach can be advantageous in ensuring the model’s output is closely aligned with key aspects of a query.

In the example by Consensus.app, Zhang et al. demonstrate how this capacity proves valuable in their example from rehabilitation medicine, where ChatGPT-4 generated targeted intervention plans by focusing on the most relevant details of a patient’s presentation.

### Key topic 2: Explaining “generic” or “textbook” responses

“Generic” or “textbook” responses arise from a model’s tendency to draw on widely represented knowledge learned from its training data (“short head knowledge”). GPT-o1 suggests that, when responding to medical queries, the model’s MLPs often rely on well-learned patterns, which can lead to standardized procedures being presented even in atypical clinical situations. This is echoed in the Consensus.app findings, indicating ChatGPT-4’s propensity to default to generalized medical knowledge.

### Key topic 3: Understanding strengths and weaknesses in differential diagnosis

With regard to key topic (3), GPT-o1 suggests that models sometimes include improbable clinical differential diagnoses due to strong correlations detected between input and training data, irrespective of the prevalence of the diagnosed disease; such base rate neglect may or may not be helpful for an accurate diagnosis. ^31^ Consensus.app mentions a lack of direct clinical experience of the models, which may refer to base rate neglect. In the example of Wu et al., ^30^ accuracy was supposedly enhanced by data augmentation using synthetic data as part of the CALLM framework, allowing better differential diagnoses based on more balanced data. Here, “synthetic data” refers to artificial training examples generated by an LLM itself – in CALLM, transcripts produced through a “patient–doctor role-playing” procedure – rather than exemplars hand-crafted by humans. This distinction matters: training models on AI-generated data carries its own risks, because recursive or large-scale use of synthetic data can degrade performance and, in the limit, lead to “model collapse”, an effect that can be triggered even by relatively small fractions of synthetic data in the training set. ^32^ Any benefit from augmentation therefore depends on careful curation, validation, and an appropriate balance of real and synthetic examples. However, skeptics may argue that true clinical complexity is difficult to replicate through synthetic data, casting doubt on the broader applicability of AI-generated simulations for real-world clinical settings. Thus, there is an evident tension between synthetic data generation and the complexity of capturing clinical “real-world” scenarios. This also underscores the critical importance of robust validation requirements, ^33^ particularly when significant decisions are to be made by an LLM.

### Key topic 4: Explaining ambiguous or contradictory responses

In addressing key topic (4), GPT-o1 links ambiguous or contradictory outputs to unclear or insufficiently specific prompts, whereas Consensus.app posits that ambiguity within the model’s training data is a contributing factor. One solution involves carefully crafted prompts designed to elicit the model’s reasoning processes, thereby mitigating confusion. Here, the CALLM framework successfully employs a “Response-Reason” prompting strategy.

### Key topic 5: Identifying hallucinations in unfamiliar scenarios

By contrast, “unfamiliar scenarios” engage a model’s “long tail” knowledge, where there is a heightened risk of hallucination because the queries may diverge significantly from what could be learned from the training set.

A case in point, discussed by Zhang et al., shows ChatGPT-4 successfully generating International Classification of Functioning (ICF) codes for a stroke patient but misreporting the lesion site. Specifically, the model accurately identified the motor dysfunction in the left hand but failed to report the lesion in the right precentral gyrus. Although the model’s output correctly flags the patient’s impaired motor function, it does not relate this impairment to its origin in disrupted motor signalling in the brain. Rather, the case illustrates a limitation of learned statistical and correlational regularities when they are not embedded in the broader causal, evidential, and clinical interpretation required in medicine. Consequently, the output treats the limitation as purely motor-related rather than reflecting the underlying neurological cause. A simpler account is that the relevant regularity – that the ICF body-structures code should refer to the primary lesion rather than its secondary consequences – was insufficiently represented in the training data (“long tail” knowledge).

This distinction, however, is crucial: if a clinical decision-support system fails to report the specific neurological lesion, it may overlook critical rehabilitation strategies that are essential for effective patient care. Consequently, interventions might miss addressing the root cause, leading to slower or less effective patient recovery.

These examples highlight the importance of recognizing not only what a model can accomplish but also where gaps in its knowledge or reasoning may lead to clinically relevant inaccuracies.

## Discussion

The implementation of AI, particularly LLMs, in healthcare will drive transformative changes in medical practice and theory while presenting significant challenges. A thorough understanding of the key ingredients of LLMs, based on their underlying architecture, including attention mechanisms and MLPs, can be particularly useful in situations where the model’s outputs are unexpected, ambiguous, or counterintuitive, necessitating critical analysis but also in routine cases where seemingly appropriate recommendations may invite automation bias.

Theoretical understanding of how LLMs process and generate information provides a conceptual framework for interpreting their outputs. ^34^ It allows clinicians and researchers to anticipate how attention mechanisms determine contextual focus, how probabilistic prediction can lead to overly generic or “textbook” responses, and why models occasionally generate contradictory or fabricated information. This type of literacy enables users to distinguish between the model’s apparent confidence and fluency and the actual reliability of its reasoning. Understanding the model’s architecture thus directly supports critical and ethical evaluation of AI-generated content in clinical contexts.

The five key topics presented here contribute to the responsible use of large language models (LLMs) in medicine. Anticipating the contextual focus in medical reasoning (key topic 1) helps users understand why certain information is extracted and prioritized by the LLM, while other aspects are neglected. Awareness of the tendency of LLMs to generate generic responses (key topic 2) draws attention to the risk of overreliance on common patterns, which may not always apply to complex, rare, or atypical cases. LLMs can quickly generate potential differential diagnoses but often show weaknesses, for instance, in aligning these diagnoses with specific epidemiological contexts. Understanding such strengths and weaknesses (key topic 3) is crucial for assessing the validity and evidential value of LLM outputs. At times, LLMs tend to produce ambiguous or contradictory responses. Identifying these limitations (key topic 4) encourages critical prompt design and iterative clarification. Finally, recognizing hallucinations in unfamiliar scenarios (key topic 5) highlights the importance of verifying outputs when the model encounters data outside its training distribution. Together, these insights show that theoretical knowledge can serve as a safeguard, helping users interpret and evaluate LLM outputs more systematically.

Knowledge of attention mechanisms and tokenization does not, by itself, ensure that clinicians are better able to detect hallucinations, unsupported clinical recommendations, or bias, and practical habits, such as checking references against the primary literature and comparing recommendations with current guidelines are more important for sure. Yet, theoretical knowledge adds explanatory value to these practical competencies, for example by indicating when generic answers (key topic 2) or hallucinations (key topic 5) are to be expected.

Two further areas of LLM literacy matter in clinical use. The first concerns what a model has learned. Its knowledge is fixed when training ends and comes from a corpus that mixes peer-reviewed evidence with non-peer-reviewed content, so outputs may lag behind current guidelines and need checking against current, reliable sources. Retrieval-augmented generation, which inserts retrieved documents into the model’s input, can make outputs more current and traceable, but the output then depends on what was retrieved and can still deviate from it, an instance related to key topic 5. ^35^ Smaller, domain-specific models can be run locally, trading breadth of capabilities for control over data. The training corpus also does not represent all populations and settings equally: some patient groups, disease patterns, treatment practices and resource levels are rare, corresponding to the long-tail knowledge in our terms. ^36^ Consequently, recommendations may not fit the local clinical context (key topic 5). The second area concerns what happens to the information that users enter. Prompts and uploaded documents may be stored, logged or used to train future models, depending on the platform. LLM applications also carry security risks of many kinds. ^8^ For example, identifiable patient data belong only in tools approved for them.

This interpretive literacy also encompasses an ethical responsibility. Understanding and identifying where LLMs fail due to inherent limitations empowers more autonomous engagement with LLMs (avoiding so-called “computer paternalism”) and helps to safeguard patient safety. ^37, 38^ It also helps to see where such failures may introduce or amplify biases, enabling clinicians to better judge when model outputs risk marginalizing minority groups or reinforcing existing prejudices. ^39^ The ethical use of LLMs should therefore not be defined solely by regulations and guidelines but is equally determined by the user’s competence in engaging with this technology. Such competence includes the ability to discern biases and misinformation that may be concealed within outputs that appear neutral and confident. ^40^

Several limitations of this work should be acknowledged. The examples analyzed were generated and selected using AI-based tools, critically examined and assessed by human authors, and represent only a small, non-systematic sample of possible cases rather than a representative or exhaustive survey. Although the use of GPT-o1 and Consensus.app provided a consistent exploratory framework, it may also have introduced biases related to model behavior and source retrieval. We also acknowledge the circularity inherent in a partially self-referential design, in which the system under study (GPT-o1) is also the source of the illustrative material. We have sought to mitigate this by treating the model’s outputs as hypotheses rather than as evidence, by cross-checking them against independent peer-reviewed studies, and by subjecting them to critical appraisal. A further, fundamental limitation concerns the partially self-referential design itself: because an LLM cannot genuinely introspect, the explanations it produces about its own “reasoning” need not correspond to the computations actually carried out inside the network, as demonstrated by mechanistic-interpretability studies. We have therefore used the model’s outputs only as a source of candidate topics and illustrations, which were then critically appraised and cross-checked against independent literature; they should not be read as evidence about the model’s internal mechanisms. ^24^ Furthermore, this study did not include feedback by external clinicians, which may further limit the generalizability of the findings. Nevertheless, by instructing LLMs and AI-assisted literature search tools to reflect on the requirements of their use in medical contexts, we applied a methodology that can serve as a powerful approach to studying AI reasoning in medicine.

Future research should build on this methodology through collaborative designs that integrate theoretical analysis with empirical testing in clinical and educational settings. Combining model introspection with user evaluation may help to further study how theoretical understanding enhances AI-literacy. Moreover, developing educational frameworks and training modules on LLM interpretability could further strengthen the competencies of students and clinicians to use AI responsibly.

It is sometimes assumed that the rapid evolution of LLMs makes it futile to build a stable understanding of their inner workings. The underlying deep-learning architecture – the transformer, with its attention mechanisms and MLP layers – has remained largely unchanged since 2017, and recent progress has come primarily from scaling up data, parameters, and compute rather than from new core mechanisms; indeed, there is ongoing debate about whether returns from further scaling are beginning to plateau. ^25^ Because the architectural principles on which our analysis rests are comparatively stable, the interpretive skills described here are likely to remain relevant even as models continue to scale. It nonetheless remains essential to meet any novel developments with a comprehensive theoretical, practical, and ethical skill set. These competencies will enable healthcare professionals to approach model outputs not as unquestionable truths but as context-dependent and probabilistic responses that warrant critical examination, thereby remaining vigilant regarding their limitations. ^7, 41^ Such a mindset fosters a reflexive awareness of how AI systems interact with clinical reasoning and decision-making, helping to preserve space for professional judgement, patient values, and situational nuance within emerging forms of human–AI collaboration. ^42, 43^ This will help maintain human oversight and ethical accountability even as models become more powerful. Ultimately, bridging medical expertise with theoretical and ethical knowledge will be necessary to ensure that AI contributes to, rather than undermines, the integrity of clinical practice.

## Conclusions

The examples analyzed in this study demonstrate that LLMs hold transformative potential in healthcare, and theoretical knowledge of their architecture and mechanisms is important for interpreting their outputs responsibly. Understanding the balance between short head and long tail knowledge, recognizing generic responses, and identifying hallucinations are important skills. Developing these competencies can support healthcare professionals in using LLMs more effectively and safely, enabling them to integrate AI technologies while maintaining appropriate oversight, mitigating risks and ensuring ethical standards.

## Data Availability

Figshare. Extended Data. https://doi.org/10.6084/m9.figshare.31493524. ^44^ This project contains the following extended data: Extended Data (All prompts, GPT-o1 outputs, and Consensus.app search results, in Supplementary Sections A and B.) Data is available under the terms of the Creative Commons Attribution 4.0 International license (CC-BY 4.0). A preliminary version of this work was made available as a preprint at https://www.preprints.org/manuscript/202510.0630/v1. ^1^ The supplementary material is available at https://doi.org/10.6084/m9.figshare.31493524. ^44^
