# Supplementary material for: Understanding the Inner Workings of Large Language Models in Medicine
Source: F1000Res. 2026 Nov 13;15:669. Originally published 2026 May 5. [Version 3] doi: 10.12688/f1000research.178855.3 (PMC13752444; doi:10.12688/f1000research.178855.3)
Supplement: Peer review history

# Reviewer report: Reviewer response for version 2

**DOI:** 10.5256/f1000research.204570.r528196

**Published:** 2026-11-10

**Authors:** Majdi Anwar Quttainah

**Affiliations:** 1 Kuwait University, Kuwait City, Kuwait

**Peer Review Evaluation Report**

**Manuscript Title:** Understanding the Inner Workings of Large Language Models in Medicine

**Journal:** F1000Research

**Recommendation:** Major Revision

**1. Objectives and Rationale**

 The manuscript's rationale—that healthcare professionals require a foundational understanding of Transformer architectures to safely interpret large language model (LLM) outputs—is relevant and timely. However, the exact boundary between purely theoretical model mechanisms and their practical utility in clinical decision-making requires further sharpening.

**Concrete Suggestions to Improve Rationale & Objectives:**

1. **Explicitly operationalize "Theoretical Knowledge":** Clearly define what minimum threshold of architectural comprehension (e.g., self-attention, token embeddings, MLP parameters) a clinician actually needs compared to general functional AI literacy.
2. **Clarify the study’s core contribution:** Explicitly state in the Introduction how this exploratory, hypothesis-generating methodology differs from standard narrative reviews or framework papers on medical AI literacy.
3. **Refine the scope of the main objective:** Rather than stating a broad aim to explore "how theoretical knowledge... can support interpretation," specify that the study seeks to generate a structured taxonomy of clinical failure modes mapped directly to underlying architectural behaviors.

**2. Novelty and Significance**

**Assessment**

 The paper presents an interesting qualitative framing by exploring whether understanding architectural components (such as multi-head attention and multi-layer perceptrons) aids in identifying failure modes like base rate neglect, generic outputs, and hallucinations.

**Critical Assessment**

- **Significance:** High relevance to medical education and AI governance. However, because the study relies on prompting GPT-o1 to self-explain architectural mechanisms and matching these to selected case studies via Consensus.app, the findings remain speculative and conceptual rather than empirically grounded.
- **Novelty:** The concept of mapping LLM limitations to architectural traits is discussed in computer science and interpretability research. The primary novelty here lies in framing these mechanisms specifically for medical decision-making and AI-assisted clinical reasoning.

**3. Relationship to Literature**

**Assessment**

 The manuscript engages with contemporary 2023–2026 literature surrounding LLM applications in healthcare, automation bias, model collapse, and mechanistic interpretability.

**Key Strengths & Gaps**

- **Strengths:** The authors appropriately cite foundational ML works (e.g., Vaswani et al.), recent studies on model collapse (Dohmatob et al.), and mechanistic interpretability (Lindsey et al.).
- **Gaps & Omissions:**
  - **Explainable AI (XAI) in Healthcare:** The manuscript overlooks broader literature on Explainable AI (XAI) and human-centered decision support, which has long studied how clinicians process algorithmic explanations.
  - **Clinical Prompt Engineering & Metacognition:** Additional synthesis is needed regarding existing frameworks for clinical prompt design and how metacognitive training in medicine aligns with algorithmic evaluation.

**4. Methodology**

**Assessment**

 The authors adopt an exploratory, qualitative approach using a self-referential design (prompting GPT-o1 to generate key topics) followed by non-systematic literature retrieval via Consensus.app.

**Critical Assessment**

- **Methodological Limitations:**
  1. **Self-Referential Circularity:** Relying on an LLM to generate insights into its own architectural failure modes poses inherent circularity risks, which the authors acknowledge but do not fully resolve.
  2. **Non-Systematic Case Selection:** Selecting only two primary studies (Zhang et al. and Wu et al.) to exemplify the five key topics limits the breadth and generalizability of the observations.
- **Conceptual Rigor:** The manuscript correctly emphasizes that LLM "introspection" is a procedural shorthand rather than true computational self-awareness. However, the link between specific architectural layers (e.g., MLPs vs. attention heads) and specific clinical error types (e.g., generic text vs. base rate neglect) remains largely illustrative rather than mechanistically proven.

**5. Results and Conclusions**

**Assessment**

 The results are categorized into five structured key topics:

1. Anticipating contextual focus in medical reasoning
2. Explaining “generic” or “textbook” responses
3. Understanding strengths and weaknesses in differential diagnosis
4. Explaining ambiguous or contradictory responses
5. Identifying hallucinations in unfamiliar scenarios

**Critical Assessment**

- **Presentation:** The topics are presented logically and present a clear taxonomy for discussing clinical AI errors.
- **Alignment of Conclusions:** The conclusions appropriately frame these five topics as preliminary competencies for healthcare professionals. However, the claim that theoretical architectural knowledge directly mitigates risk in clinical practice remains a hypothesis that requires future empirical testing with real clinicians.

**6. Statistical Reporting and Analysis**

**Assessment**

 This manuscript is a qualitative, conceptual report and contains no quantitative empirical data, formal statistical tests, sample size calculations, $P$-values, or confidence intervals.

**Suggestions for Statistical / Empirical Improvements:**

1. **Acknowledge the lack of empirical validation:** Explicitly clarify in the Methods that no statistical hypotheses were tested.
2. **Propose quantitative metrics for future studies:** Suggest specific metrics for follow-up user trials (e.g., diagnostic accuracy rates, time-to-decision, automation bias reduction rates measured via odds ratios or $A sequence$ score metrics).
3. **Statistician Review:** A formal review by a statistician is **not required** for the present manuscript version, given its non-quantitative nature.

**7. Implications for Research, Practice, and Society**

- **Practice:** Provides a structured framework for clinicians to critically evaluate LLM outputs rather than treating them as black-box systems.
- **Research:** Serves as a foundational starting point for designing user studies to measure whether teaching transformer mechanics to medical students measurably reduces automation bias.
- **Society & Ethics:** Directly addresses automation bias, paternalism in AI systems, and patient safety by emphasizing human accountability and critical oversight.

**8. Quality of Communication**

- **Readability and Tone:** The writing is clear, articulate, and well-structured. The terminology used in computer science (e.g., short head vs. long tail knowledge, synthetic data, multi-head attention) and medicine is handled accurately.
- **Clarity of Expression:** Key concepts and qualifiers regarding "mentalistic" language (e.g., reasoning, focus, knowledge) are explicitly defined and appropriately qualified throughout the manuscript.

**Final Recommendation**

**Major Revision**

 The manuscript provides valuable conceptual insights into medical AI literacy. To meet publication standards, the authors should address the non-systematic nature of their case study selection, clarify the empirical boundaries of their claims, and explicitly outline a research roadmap for testing these hypotheses in prospective clinical user trials.

Is the work clearly and accurately presented and does it cite the current literature?

Partly

If applicable, is the statistical analysis and its interpretation appropriate?

Not applicable

Are all the source data underlying the results available to ensure full reproducibility?

No

Is the study design appropriate and is the work technically sound?

Partly

Are the conclusions drawn adequately supported by the results?

Partly

Are sufficient details of methods and analysis provided to allow replication by others?

Partly

Reviewer Expertise:

The core premise of the manuscript is compelling and highly relevant to strategic HRM and organizational governance. However, to meet the standards of publication in a leading academic journal, the authors must undertake a major revision focusing on:Clarifying the unit of analysis and case study design within the qualitative methodology.Deepening the qualitative presentation with richer narrative data beyond brief single-line quotes.Expanding theoretical integration with foundational literature on algorithmic control and regulatory frameworks.Resolving sampling bias concerns and internal numerical discrepancies.

I confirm that I have read this submission and believe that I have an appropriate level of expertise to confirm that it is of an acceptable scientific standard, however I have significant reservations, as outlined above.

# Reviewer report: Reviewer response for version 2

**DOI:** 10.5256/f1000research.204570.r532954

**Published:** 2026-10-26

**Authors:** Devi Prasad Mohapatra

**Affiliations:** 1 Jawaharlal Institute of Postgraduate Medical Education and Research, Pondicherry, Puducherry, India

I liked reading the manuscript and it is quite relevant to the present scenario. 

 The objective of the study is interesting and relevant to the growing use of large language models (LLMs) in clinical practice. The authors aim to explore whether a theoretical understanding of LLM architecture and internal mechanisms can help physicians better interpret and critically evaluate model-generated content.

 One particularly interesting statement in the manuscript is that *“an LLM does not have direct access to its own internal computations.”* This highlights an important limitation of these models: although they can generate plausible explanations for their responses, such explanations do not necessarily represent the actual computational processes responsible for generating those responses.

 LLMs primarily operate by learning statistical and contextual relationships within large volumes of training data and generating outputs based on learned probability distributions. Although they can demonstrate sophisticated language understanding and reasoning capabilities, their underlying mechanisms differ from those of human cognition. This distinction has been discussed by Cuskley et al. (2024) ^1^, who examined the limitations of LLMs as models of human language and cognition.

 The authors have identified five key topics related to interpretability, readability, and clinical applications of LLMs. They have used ChatGPT to identify these topics and  Consensus to retrieve relevant peer-reviewed literature and prepare a synopsis of each topic. The methodology described facilitates a conceptual exploration of the subject. Demonstrating an improvement in physicians' interpretive abilities would require an appropriate empirical evaluation involving clinicians (which the authors have acknowledged in their study limitations).

 Also, I feel that a physician may understand attention mechanisms and tokenization without necessarily becoming better at detecting hallucinations, identifying unsupported clinical recommendations, or recognizing bias, while physicians can learn to evaluate AI-generated clinical information effectively without detailed knowledge of neural network mathematics also.

 As I understand it, the underlying objective of this exercise is to explore whether greater AI literacy, particularly an understanding of LLM architecture and functioning, could help physicians interpret, critically evaluate, and appropriately use LLM-generated information in clinical practice.

 The authors have presented their hypothesis appropriately and I agree with this. 

 Beyond the five key topics identified through ChatGPT, I feel that there are several additional areas of LLM literacy that are particularly relevant to physicians and deserve consideration.

**1. Training data, currency of medical knowledge, and information retrieval**

 An important aspect of LLM literacy is understanding the nature and limitations of the data used to train these models. In a rapidly evolving field such as medicine, information derived from training datasets may become outdated as newer evidence, guidelines, and treatment recommendations emerge. Furthermore, the inclusion of non-peer-reviewed medical information and other internet-based material in training datasets raises concerns regarding the reliability and evidence quality of generated responses. Physicians should therefore understand the importance of verifying AI-generated information against current, reliable medical sources.

 Some knowledge of small language models and retrieval-augmented generation (RAG), including their potential advantages and limitations, would also be relevant for physicians as part of LLM literacy

**2. Bias and representativeness of training data**

 The potential for bias arising from the composition of training datasets needs to be considered. The sources and demographic representativeness of the data used to train many commercial LLMs are not fully transparent. This is particularly relevant from a global healthcare perspective, where disease patterns, treatment practices, resource availability, and patient characteristics may vary considerably across geographic regions and populations. Physicians should be aware that recommendations generated by LLMs may not always be appropriate for their local clinical context.

**3. Handling of information submitted through prompts and uploaded documents**

 Physicians also need to understand how information submitted to LLM-based applications is processed and potentially retained or used. User-provided information may be stored, logged, or subsequently used for model improvement, depending on the platform and its data policies. This will be important in clinical practice, where physicians may inadvertently submit identifiable patient information, clinical records, or other confidential material.

**4. Security and privacy risks associated with LLM applications**

 Security and privacy implications of using LLM-based systems in clinical practice needs to be understood. While the internal functioning of these models may not be fully interpretable, the handling and protection of submitted information are primarily matters of application security and data governance. Risks like unauthorized access to stored information, inadvertent disclosure of confidential patient data, and vulnerabilities associated with third-party applications or integrations have to be kept in mind.

 While understanding the underlying mechanisms may help physicians appreciate the capabilities and limitations of these systems, practical competencies such as identifying hallucinations, verifying references, recognizing bias, and evaluating the clinical applicability of generated responses may be equally important.

 References 

 Refer (1)

 AI Disclosure: The language of the report has been refined using GPT 6 (ChatGPT, Open AI). The scientific observations, assessments, and recommendations are entirely my own, and I have reviewed and approved the final content.

Is the work clearly and accurately presented and does it cite the current literature?

Partly

If applicable, is the statistical analysis and its interpretation appropriate?

Not applicable

Are all the source data underlying the results available to ensure full reproducibility?

Yes

Is the study design appropriate and is the work technically sound?

Partly

Are the conclusions drawn adequately supported by the results?

Partly

Are sufficient details of methods and analysis provided to allow replication by others?

Yes

Reviewer Expertise:

Medical education, faculty development, artificial intelligence, large language models in medicine and plastic surgery

I confirm that I have read this submission and believe that I have an appropriate level of expertise to confirm that it is of an acceptable scientific standard.

## References

[1] : The Limitations of Large Language Models for Understanding Human Language and Cognition. *Open Mind* .2024;8: 10.1162/opmi_a_001601058-1083 10.1162/opmi_a_00160 PMC1137097039229609

# Response

**Published:** 2026-10-30

**Authors:** Georg Fuellen

**Affiliations:** IBIMA, Rostock University Medical Center, Rostock, Germany

We thank Reviewer 3 for the constructive assessment of our Brief Report and for identifying further aspects of LLM literacy relevant to physicians. We have incorporated the suggestions within the scope of the present conceptual article by making a focused clarification in the Methods, adding two paragraphs to the Discussion, and adding three references (Cuskley et al., Lewis et al., and Omiye et al.). The five key topics and the exploratory design remain unchanged.

 These additions also complement, in part, revisions made in response to the other reviewers, especially by more clearly distinguishing LLM-generated explanations from human understanding and by emphasizing the need for independent clinical appraisal. Below, we quote the pertinent passages from Reviewer 3 verbatim and respond point by point.

**> 1. LLM mechanisms and human cognition**

 > LLMs primarily operate by learning statistical and contextual relationships within large volumes of training data and generating outputs based on learned probability distributions. Although they can demonstrate sophisticated language understanding and reasoning capabilities, their underlying mechanisms differ from those of human cognition. This distinction has been discussed by Cuskley et al. (2024)1, who examined the limitations of LLMs as models of human language and cognition.

**Response: **We agree. In the Methods subsection “Large Language Models (LLMs),” we now explicitly distinguish LLM mechanisms from human cognition, even when the generated outputs resemble human reasoning. We now cite Cuskley et al. (2024). This complements our existing qualifications concerning terms such as “knowledge,” “reasoning,” and “understanding.”

**> 2. Theoretical knowledge and practical evaluation skills**

 > Also, I feel that a physician may understand attention mechanisms and tokenization without necessarily becoming better at detecting hallucinations, identifying unsupported clinical recommendations, or recognizing bias, while physicians can learn to evaluate AI-generated clinical information effectively without detailed knowledge of neural network mathematics also.

**Response: **We agree that knowledge of model architecture does not itself establish improved clinical evaluation performance. The first new paragraph of the Discussion now makes this distinction explicit and stresses practical verification of references against primary literature and recommendations against current clinical guidelines. Our argument is that theoretical knowledge can complement these practical competencies, for example by drawing attention to the risk of generic responses or hallucinations (key topics 2 and 5), not replace them.

**> 3. Training-data currency, RAG and smaller models**

 > Training data, currency of medical knowledge, and information retrieval

 An important aspect of LLM literacy is understanding the nature and limitations of the data used to train these models. In a rapidly evolving field such as medicine, information derived from training datasets may become outdated as newer evidence, guidelines, and treatment recommendations emerge. Furthermore, the inclusion of non-peer-reviewed medical information and other internet-based material in training datasets raises concerns regarding the reliability and evidence quality of generated responses. Physicians should therefore understand the importance of verifying AI-generated information against current, reliable medical sources.

 Some knowledge of small language models and retrieval-augmented generation (RAG), including their potential advantages and limitations, would also be relevant for physicians as part of LLM literacy

**Response: **We have added these issues to the second new Discussion paragraph. It emphasizes the possible lack of currency and mixed evidential quality of training materials, the importance of checking outputs against current reliable medical sources, and the potential and limitations of RAG and smaller models. We also added Lewis et al. (2020).

**> 4. Bias and representativeness of training data**

 > Bias and representativeness of training data

 The potential for bias arising from the composition of training datasets needs to be considered. The sources and demographic representativeness of the data used to train many commercial LLMs are not fully transparent. This is particularly relevant from a global healthcare perspective, where disease patterns, treatment practices, resource availability, and patient characteristics may vary considerably across geographic regions and populations. Physicians should be aware that recommendations generated by LLMs may not always be appropriate for their local clinical context.

**Response: **The second new Discussion paragraph now addresses bias and representativeness explicitly. It connects this concern to the “long-tail” knowledge discussed in the manuscript and cautions that recommendations may not fit a local clinical context. Here, we added Omiye et al. (2023).

**> 5. Information submitted in prompts and documents**

 > Handling of information submitted through prompts and uploaded documents

 Physicians also need to understand how information submitted to LLM-based applications is processed and potentially retained or used. User-provided information may be stored, logged, or subsequently used for model improvement, depending on the platform and its data policies. This will be important in clinical practice, where physicians may inadvertently submit identifiable patient information, clinical records, or other confidential material.

**Response: **The second new Discussion paragraph now explains the risks noted by the reviewer. It emphasizes that identifiable patient information should be entered only into systems approved for that purpose.

**> 6. Privacy and application security**

 > Security and privacy risks associated with LLM applications

 Security and privacy implications of using LLM-based systems in clinical practice needs to be understood. While the internal functioning of these models may not be fully interpretable, the handling and protection of submitted information are primarily matters of application security and data governance. Risks like unauthorized access to stored information, inadvertent disclosure of confidential patient data, and vulnerabilities associated with third-party applications or integrations have to be kept in mind.

**Response: **We now briefly flag the security risks associated with LLM applications and the need to protect patient information in the second new Discussion paragraph. We agree that these concerns primarily relate to application security and data governance, rather than to mechanistic interpretability. We have kept the addition concise to preserve the scope of the Brief Report.

**> 7. The complementary role of practical competencies**

 > While understanding the underlying mechanisms may help physicians appreciate the capabilities and limitations of these systems, practical competencies such as identifying hallucinations, verifying references, recognizing bias, and evaluating the clinical applicability of generated responses may be equally important.

**Response: **We agree. This is the central point of the first new Discussion paragraph: theoretical knowledge may provide explanatory value, but it does not replace verification against clinical evidence, bias awareness, or assessment of clinical applicability. Our Brief Report remains exploratory; it does not claim to establish empirically that greater architectural knowledge improves physicians’ performance.

 We thank Reviewer 3 again for these helpful suggestions. The revisions broaden the clinical perspective while preserving the paper’s five-topic framework and its exploratory, hypothesis-generating purpose.

 New references added:

1. Cuskley C, Woods R, Flaherty, M: The limitations of large language models for understanding human language and cognition. *Open Mind*. 2024;8:1058-1083. 10.1162/opmi_a_00160
2. Lewis P, Perez E, Piktus A, et al.: Retrieval-augmented generation for knowledge-intensive NLP tasks. *Adv Neural Inf Process Syst*. 2020;33:9459–9474.
3. Omiye JA, Lester JC, Spichak S, et al.: Large language models propagate race-based medicine. *NPJ Digit Med*. 2023;6:195. 10.1038/s41746-023-00939-z

# Reviewer report: Reviewer response for version 1

**DOI:** 10.5256/f1000research.197293.r487509

**Published:** 2026-06-04

**Authors:** Craig S Webster

**Affiliations:** 1 The University of Auckland, Auckland, Auckland, New Zealand

This is an interesting paper that I enjoyed reading. It makes a number of key distinctions which I think are in fact important for the users of LLMs to better understand when they should be trusted and when users need to be more critical. This is an important and practical concern for clinicians using AI-based tools in healthcare, and so from this perspective I think the paper has merit. I like the short-head and long-tail distinction and how this relates to the risk of hallucination – this is of real interest to clinicians who are not technical experts in LLM technology, but need to know how best to use them. The five key topics will also be of interest to clinicians. However, these positives aside, there are a number of areas where the language in the paper needs to be tightened, as below.

 Page 3, 2 ^nd^ paragraph: you mention that LLMs may on occasion work best without human intervention. I think some unpacking of what you mean by this is needed, as most authorities believe that AI use in healthcare must always be supervised by humans. Humans are the ultimate sense makers and decision makers, since the AI has no actual understanding of the tasks it performs or their consequences.

 Page 4: You mention synthetic data augmentation to fill gaps in training data sets. It was unclear to me what you meant by synthetic data – do you mean exemplar data made up by humans, or data generated by other AI systems? Synthetic data generated by other AI systems has been shown to reduce the performance of LLMs, even to the extent of so-called model collapse, and even with surprisingly small amounts of synthetic data in the training data set. Hence, I think you need to be clearer about what you mean by synthetic data, and also to explain how you might avoid performance decline if you are using such data, or at least mention the risks. See: Elvis Dohmatob, Yunzhen Feng, Arjun Subramonian and Julia Kempe. Strong Model Collapse, arXiv, 2024. https://arxiv.org/abs/2410.04840

 One of the central problems of asking an LLM to explain itself, or to “introspect” is that all LLM responses are simply text outputs designed to have the highest probability of being correct given the training data and the prompt. Hence the LLM makes no distinction between an “introspective” prompt and one asking a more general inquiry. LLMs have no sense of self, or awareness of what they are doing, hence cannot actually introspect in the way we understand it. Often an LLM will give you the textbook answer to some task it just performed because that method was in its training data, but when you inspect the activity of the neural network itself using something like a mechanistic interpretability approach, you find that what it actually did was nothing like what it just claimed. It may claim some tidy, logical method, but what is actually going on inside the network is typically highly complicated, probabilistic, and essentially unintelligible – it just happens to get the right answer most of the time. See: On the Biology of a Large Language Model, https://transformer-circuits.pub/2025/attribution-graphs/biology.html

 I think you need to mention this gap between what the LLM claims it is doing, and what is actually going on in the network.

 Page 6, key topic 5: I think this is a very interesting discussion, and it underscores the point I was making in my previous comment – the LLM has no understanding of what it is doing. More critically for the use of LLMs in medicine, the rules that the LLM has extracted from the training data through statistical inference are not equivalent to evidence-based medicine or the causal theories of disease! And this is a key point that many clinicians do not appreciate. This is why it doesn’t make the connection between symptoms and underlying causes, it has only a probabilistic or correlational model, not a causal one. I think you need to be very careful about using words like “understand”, “knowledge”, “reasoning” and indeed “meta-knowledge” when describing what the LLMs is doing – as technically, it has none of these things (although it may appear to have them). For a discussion of these key distinctions, which are highly relevant to medicine, see: Webster, C. S. (2025). Natural and artificial intelligence – the psychotechnical agenda of the 21st century. Journal of Psychology and AI, 1(1). https://doi.org/10.1080/29974100.2025.2491445

 Page 8, 5 ^th^ paragraph: You make a claim about the rapid evolution of LLMs making it hard to understand their inner workings. Actually, the underlying technology of deep learning models hasn’t changed much in years, what we have seen recently is a scaling up of this technology – whether it continues to scale up or not is a question of debate – although recent evidence suggests that performance of these models is plateauing. However, given that the underlying technology of deep learning remains the same in all these models, the aim of your paper remains relevant, despite the results of scaling up – and I think you should make this point in discussion.

Is the work clearly and accurately presented and does it cite the current literature?

Partly

If applicable, is the statistical analysis and its interpretation appropriate?

Yes

Are all the source data underlying the results available to ensure full reproducibility?

Yes

Is the study design appropriate and is the work technically sound?

Yes

Are the conclusions drawn adequately supported by the results?

Yes

Are sufficient details of methods and analysis provided to allow replication by others?

Yes

Reviewer Expertise:

Neuropsychology, clinical education, system redesign, artificial intelligence

I confirm that I have read this submission and believe that I have an appropriate level of expertise to confirm that it is of an acceptable scientific standard, however I have significant reservations, as outlined above.

# Response

**Published:** 2026-06-21

**Authors:** Georg Fuellen

**Affiliations:** IBIMA, Rostock University Medical Center, Rostock, Germany

**Response to Reviewers**

 We thank both reviewers for their careful and constructive reading of our Brief Report. We are encouraged that Dr. Webster found the work interesting and valued the short-head/long-tail distinction and the five key topics, and that Dr. Kıyak considered the underlying question important. The reviewers' comments converge on a single, fair theme: that the language of the first version sometimes claimed more than an exploratory study can support, and that several statements about what the model "understands" or "knows" needed to be tightened and qualified. We agree, and we have revised the manuscript accordingly.

 In brief, the revision makes four kinds of changes. (1) We reframe the contribution explicitly as exploratory and hypothesis-generating rather than as empirical evidence that theoretical knowledge improves clinicians' performance. (2) We replace "validation" language with "cross-checking/illustration" throughout, and we state that Consensus.app was used to retrieve corroborating literature, not to validate model-generated themes. (3) We add explicit caveats that an LLM cannot genuinely introspect, that mentalistic terms ("knowledge", "reasoning", "meta-knowledge"), where retained, are used in a careful and qualified sense to describe  the results of statistical/correlational operations, and that the model's learned correlations do not necessarily reflect causal or evidence-based medicine. (4) We clarify the "without human intervention" claim, define "synthetic data" and add the model-collapse risk, and nuance the "rapid evolution" claim. 

 Below we respond to each point in turn.

**Reviewer 2** — Craig S. Webster (Approved with reservations)

 2.1 "Best results without human intervention" (p.3, 2nd paragraph).

**Reviewer comment:** "Page 3, 2nd paragraph: you mention that LLMs may on occasion work best without human intervention. I think some unpacking of what you mean by this is needed, as most authorities believe that AI use in healthcare must always be supervised by humans. Humans are the ultimate sense makers and decision makers, since the AI has no actual understanding of the tasks it performs or their consequences."

**Response:** We have unpacked this. The cited evidence concerns narrowly defined benchmark tasks on which unaided model accuracy can match or exceed human–AI teams (e.g., the meta-analysis of Vaccaro et al.); it is a descriptive performance observation, not a normative claim about clinical autonomy. We now say so explicitly and reaffirm that clinical use must remain human-supervised.

 Change — Introduction (2nd paragraph):

**Original: **"This problematic development ('automation bias') can be further reinforced by evidence that, in certain tasks, LLMs already deliver the best results without human intervention."

**Revised:** "This problematic development ('automation bias') can be further reinforced by evidence that, on certain benchmark tasks, unaided LLMs can match or even exceed the accuracy of human–AI teams (Goh et al., 2024; McDuff et al. 2025). We stress that this is a narrow, task-specific performance observation and not a normative claim about clinical autonomy: in healthcare, LLM outputs must remain under human supervision, because the model has no human-like understanding of the tasks it performs or of their consequences, and the clinician remains the responsible sense-maker and decision-maker. "

 2.2 Define "synthetic data"; note model-collapse risk (p.4).

**Reviewer comment:** "Page 4: You mention synthetic data augmentation to fill gaps in training data sets. It was unclear to me what you meant by synthetic data – do you mean exemplar data made up by humans, or data generated by other AI systems? Synthetic data generated by other AI systems has been shown to reduce the performance of LLMs, even to the extent of so-called model collapse, and even with surprisingly small amounts of synthetic data in the training data set. Hence, I think you need to be clearer about what you mean by synthetic data, and also to explain how you might avoid performance decline if you are using such data, or at least mention the risks. See: Elvis Dohmatob, Yunzhen Feng, Arjun Subramonian and Julia Kempe. Strong Model Collapse, arXiv, 2024. https://arxiv.org/abs/2410.04840"

**Response:** We have clarified that, in the CALLM example, "synthetic data" means AI-generated training examples (LLM "patient–doctor role-playing" transcripts), not human-crafted exemplars, and we now flag the model-collapse risk and cite Dohmatob et al. (2024), noting that benefit depends on careful curation, validation, and an appropriate real/synthetic balance.

 Change — Results, Key topic 3 (added after "… based on more balanced data."): "Here, 'synthetic data' refers to artificial training examples generated by an LLM itself — in CALLM, transcripts produced through a 'patient–doctor role-playing' procedure — rather than exemplars hand-crafted by humans. This distinction matters: training models on AI-generated data carries its own risks, because recursive or large-scale use of synthetic data can degrade performance and, in the limit, lead to 'model collapse,' an effect that can be triggered even by relatively small fractions of synthetic data in the training set (Dohmatob et al., 2024). Any benefit from augmentation therefore depends on careful curation, validation, and an appropriate balance of real and synthetic examples."

 2.3 The gap between what an LLM claims it does and what it actually does.

**Reviewer comment:** "LLMs have no sense of self, or awareness of what they are doing, hence cannot actually introspect in the way we understand it. Often an LLM will give you the textbook answer to some task it just performed because that method was in its training data, but when you inspect the activity of the neural network itself using something like a mechanistic interpretability approach, you find that what it actually did was nothing like what it just claimed. It may claim some tidy, logical method, but what is actually going on inside the network is typically highly complicated, probabilistic, and essentially unintelligible – it just happens to get the right answer most of the time. See: On the Biology of a Large Language Model, https://transformer-circuits.pub/2025/attribution-graphs/biology.html"

**Response: **We have added a terminological note where the architecture is introduced, clarifying that terms such as “understanding,” “reasoning,” and “meta-knowledge” are not used to attribute human-like cognition, intentionality, or clinical responsibility to the model. Rather, where retained, they refer to functional roles within a broader human–AI epistemic process. The revised text now frames the issue more precisely as a limitation of learned statistical and correlational patterns. The Methods now state that the model cannot genuinely introspect and that its self-descriptions need not match its actual computation, citing Lindsey et al. (2025) (see the Methods–Overview change quoted under 1.1). To the Limitations we added the corresponding caveat.

 Change — Discussion, Limitations (added): "A further, fundamental limitation concerns the partially self-referential design itself: because an LLM cannot genuinely introspect, the explanations it produces about its own 'reasoning' need not correspond to the computations actually carried out inside the network, as demonstrated by mechanistic-interpretability studies. We have therefore used the model's outputs only as a source of candidate topics and illustrations, which were then critically appraised and cross-checked against independent literature; they should not be read as evidence about the model's internal mechanisms (Lindsey et al. (2025))."

 2.4 Careful use of "understand", "knowledge", "reasoning", "meta-knowledge"; correlation vs causation (p.6, key topic 5).

**Reviewer comment:** "Page 6, key topic 5: I think this is a very interesting discussion, and it underscores the point I was making in my previous comment – the LLM has no understanding of what it is doing. More critically for the use of LLMs in medicine, the rules that the LLM has extracted from the training data through statistical inference are not equivalent to evidence-based medicine or the causal theories of disease! And this is a key point that many clinicians do not appreciate. This is why it doesn’t make the connection between symptoms and underlying causes, it has only a probabilistic or correlational model, not a causal one. I think you need to be very careful about using words like “understand”, “knowledge”, “reasoning” and indeed “meta-knowledge” when describing what the LLMs is doing – as technically, it has none of these things (although it may appear to have them). For a discussion of these key distinctions, which are highly relevant to medicine, see: Webster, C. S. (2025). Natural and artificial intelligence – the psychotechnical agenda of the 21st century. Journal of Psychology and AI, 1(1). https://doi.org/10.1080/29974100.2025.2491445"

**Response:** We agree that mentalistic terms require careful qualification when used in relation to LLMs, especially terms such as “understanding” that may imply human-like comprehension. Where terms such as “reasoning” are retained, they are used in a qualified sense: not to attribute human-like mental states to the model, but to denote the functional role of statistically generated outputs within distributed, hybrid, and human-interpreted epistemic practices. We cite Webster (2025) to acknowledge the importance of distinguishing natural and artificial intelligence. We have also rewritten the “Key topic 5” passage so that it no longer attributes literal "understanding" or "meta-knowledge" to the model and instead frames the failure as correlational rather than causal.

 Change — Methods, Large Language Models (added): “Throughout this paper, terms such as ‘attention,’ ‘focus,’ ‘knowledge,’ ‘reasoning,’ and ‘meta-knowledge’ are used only in a careful and qualified sense. They do not attribute human-like mental states, beliefs, intentionality, or clinical responsibility to the model. Natural and artificial intelligence must be distinguished however (Webster, 2025), and here we refer to the functional role of statistically generated outputs within distributed, hybrid, and human-interpreted epistemic practices. We avoid attributing literal ‘understanding’ to LLMs: the model does not possess understanding or causal models of disease in the human sense; the regularities it extracts by statistical inference cannot be equivalent to executing evidence-based medicine or employing causal theories of disease."

 Change — Results, Key topic 5:

**Original: **"Although the model recognizes that the patient's motor function is impaired, it does not appear to understand that this impairment originates from disrupted motor signals in the brain. As a result, the model interprets the limitation as purely motor-related rather than addressing the underlying neurological cause. Alternatively, we suggest that it may miss the meta-knowledge that the lesion site to be reported here shall refer to the underlying primary lesion, not its secondary consequences."

**Revised:** "Although the model's output correctly flags the patient's impaired motor function, it does not relate this impairment to its origin in disrupted motor signalling in the brain. Rather, the case illustrates a limitation of learned statistical and correlational regularities when they are not embedded in the broader causal, evidential, and clinical interpretation required in medicine.  Consequently, the output treats the limitation as purely motor-related rather than reflecting the underlying neurological cause. A simpler account is that the relevant regularity — that the ICF body-structures code should refer to the primary lesion rather than its secondary consequences — was insufficiently represented in the training data ('long tail' knowledge)."

 2.5 "Rapid evolution" of LLMs vs. stable underlying technology (p.8, 5th paragraph).

**Reviewer comment: **"Page 8, 5th paragraph: You make a claim about the rapid evolution of LLMs making it hard to understand their inner workings. Actually, the underlying technology of deep learning models hasn’t changed much in years, what we have seen recently is a scaling up of this technology – whether it continues to scale up or not is a question of debate – although recent evidence suggests that performance of these models is plateauing. However, given that the underlying technology of deep learning remains the same in all these models, the aim of your paper remains relevant, despite the results of scaling up – and I think you should make this point in discussion."

**Response: **This is a helpful reframing that actually strengthens our argument, and we have adopted it. We now note that the transformer architecture has been largely stable since 2017, that recent gains stem mainly from scaling (with an open debate about plateauing returns), and that the stability of these architectural principles is precisely what makes the interpretive skills we describe durable.

 Change — Discussion (penultimate paragraph):

**Original:** "The rapid evolution of LLMs makes it increasingly challenging to continuously adapt our understanding of their complexity. Nevertheless, it is essential to meet this development with a comprehensive theoretical, practical, and ethical skill set."

**Revised: **"It is sometimes assumed that the rapid evolution of LLMs makes it futile to build a stable understanding of their inner workings. The underlying deep-learning architecture — the transformer, with its attention mechanisms and MLP layers — has remained largely unchanged since 2017 (Vaswani et al., 2017), and recent progress has come primarily from scaling up data, parameters, and compute rather than from new core mechanisms; indeed, there is ongoing debate about whether returns from further scaling are beginning to plateau. Because the architectural principles on which our analysis rests are comparatively stable, the interpretive skills described here are likely to remain relevant even as models continue to scale. It nonetheless remains essential to meet any novel developments with a comprehensive theoretical, practical, and ethical skill set."

 New references added

 Goh E, Gallo R, Hom J, et al. Large Language Model Influence on Diagnostic Reasoning: A Randomized Clinical Trial. JAMA Netw Open. 2024;7(10):e2440969-e2440969. doi:10.1001/jamanetworkopen.2024.40969

 McDuff D, Schaekermann M, Tu T, et al. Towards accurate differential diagnosis with large language models. Nature.2025;642(8067):451-457. doi:10.1038/s41586-025-08869-4

 Lindsey J, Gurnee W, Ameisen E, et al. On the Biology of a Large Language Model. Transformer Circuits Thread. 2025. Available from: https://transformer-circuits.pub/2025/attribution-graphs/biology.html

 Webster CS. Natural and artificial intelligence – the psychotechnical agenda of the 21st century. Journal of Psychology and AI. 2025;1(1). doi:10.1080/29974100.2025.2491445.

 Dohmatob E, Feng Y, Subramonian A, Kempe J. Strong model collapse. In: International Conference on Learning Representations; 2025:15656-15691. Available from: https://proceedings.iclr.cc/paper_files/paper/2025/file/284afdc2309f9667d2d4fb9290235b0c-Paper-Conference.pdf

 We believe these revisions address the reviewers' concerns while preserving the paper's purpose as an exploratory, hypothesis-generating Brief Report. We are grateful for feedback that has materially improved the clarity and the epistemic calibration of the manuscript.

# Reviewer report: Reviewer response for version 1

**DOI:** 10.5256/f1000research.197293.r482232

**Published:** 2026-05-18

**Authors:** Yavuz Selim Kıyak

**Affiliations:** 1 Gazi University, Ankara, Turkey

The manuscript focuses on an important possible use of LLMs. It is if theoretical knowledge of LLM architecture can help doctors better interpret AI-generated outputs. However, the current version includes several methodological concerns.

 The main methodological concern is the self-referential design. The authors used GPT-o1 to generate examples of how understanding LLMs can help evaluate LLM outputs. This leads to a circularity problem. The problem is that the tool being examined also becomes a source of evidence for the claims being developed. This might be seen acceptable for exploratory idea generation but it is not sufficiently strong for a paper.

 Another concern is overclaiming contribution. The introduction states that the study addresses a gap in empirical work but the current approach in the manuscript does not include clinicians, users, performance outcomes, comparison groups, or observed decision-making. Therefore, the study cannot show that theoretical knowledge actually improves clinicians’ ability to evaluate LLM outputs.

 The authors also used Consensus.app as a validation tool. This is another important concern. An AI-assisted literature search platform can help identify relevant papers but it does not itself validate GPT-generated themes. Another concern is the limited and selective evidence base. Only a small number of “especially illustrative” studies are used to support the final themes. There is also a need for the language of the introduction to be softened. Terms such as “validated” gives a signal like strong evidence.

Is the work clearly and accurately presented and does it cite the current literature?

Partly

If applicable, is the statistical analysis and its interpretation appropriate?

Not applicable

Are all the source data underlying the results available to ensure full reproducibility?

Partly

Is the study design appropriate and is the work technically sound?

No

Are the conclusions drawn adequately supported by the results?

No

Are sufficient details of methods and analysis provided to allow replication by others?

No

Reviewer Expertise:

medical education, large language models

I confirm that I have read this submission and believe that I have an appropriate level of expertise to state that I do not consider it to be of an acceptable scientific standard, for reasons outlined above.

# Response

**Published:** 2026-06-21

**Authors:** Georg Fuellen

**Affiliations:** IBIMA, Rostock University Medical Center, Rostock, Germany

**Response to Reviewers**

 We thank both reviewers for their careful and constructive reading of our Brief Report. We are encouraged that Dr. Webster found the work interesting and valued the short-head/long-tail distinction and the five key topics, and that Dr. Kıyak considered the underlying question important. The reviewers' comments converge on a single, fair theme: that the language of the first version sometimes claimed more than an exploratory study can support, and that several statements about what the model "understands" or "knows" needed to be tightened and qualified. We agree, and we have revised the manuscript accordingly.

 In brief, the revision makes four kinds of changes. (1) We reframe the contribution explicitly as exploratory and hypothesis-generating rather than as empirical evidence that theoretical knowledge improves clinicians' performance. (2) We replace "validation" language with "cross-checking/illustration" throughout, and we state that Consensus.app was used to retrieve corroborating literature, not to validate model-generated themes. (3) We add explicit caveats that an LLM cannot genuinely introspect, that mentalistic terms ("knowledge", "reasoning", "meta-knowledge"), where retained, are used in a careful and qualified sense to describe  the results of statistical/correlational operations, and that the model's learned correlations do not necessarily reflect causal or evidence-based medicine. (4) We clarify the "without human intervention" claim, define "synthetic data" and add the model-collapse risk, and nuance the "rapid evolution" claim. 

 Below we respond to each point in turn.

**Reviewer 1 **— Yavuz Selim Kıyak (Not approved)

 1.1 Self-referential design / circularity.

**Reviewer comment:** "The main methodological concern is the self-referential design. The authors used GPT-o1 to generate examples of how understanding LLMs can help evaluate LLM outputs. This leads to a circularity problem. The problem is that the tool being examined also becomes a source of evidence for the claims being developed. This might be seen as acceptable for exploratory idea generation but it is not sufficiently strong for a paper."

**Response: **We have explicitly addressed the potential problem of circularity. Our intent was not to use GPT-o1 as an arbiter of truth but rather in an exploratory manner: the model's outputs are the _object_ of analysis, providing candidate topics and illustrations that the human authors then appraised critically and cross-checked against independent peer-reviewed literature. We have added statements to the Methods and to the Limitations that name the circularity and describe that we therefore treat outputs as hypotheses, and do independent cross-checking, and critical appraisal. We have also described the design as "exploratory and hypothesis-generating" throughout.

 Change — Methods, Overview:

**Original:** "We conducted an exploratory analysis using AI-assisted tools in a form of model introspection and self-reflection, respectively."

**Revised:** “We conducted an exploratory analysis using AI-assisted tools in a form of model ‘introspection’. This term is used here as a procedural shorthand for prompting the model to generate explanations of its own outputs: an LLM does not have direct access to its own internal computations, and its explanations of its outputs should not be treated as genuine introspective reports. Mechanistic-interpretability work has shown that the procedure a model reports can diverge substantially from the computation it actually performs (Lindsey, 2025). We therefore treat the model’s outputs throughout as generated hypotheses to be examined critically by the human authors.”

 Change — Discussion, Limitations (added): "We also acknowledge the circularity inherent in a partially self-referential design, in which the system under study (GPT-o1) is also the source of the illustrative material. We have sought to mitigate this by treating the model's outputs as hypotheses rather than as evidence, by cross-checking them against independent peer-reviewed studies, and by subjecting them to critical appraisal."

 1.2 Overclaiming the contribution.

**Reviewer comment:** "The introduction states that the study addresses a gap in empirical work but the current approach in the manuscript does not include clinicians, users, performance outcomes, comparison groups, or observed decision-making. Therefore, the study cannot show that theoretical knowledge actually improves clinicians’ ability to evaluate LLM outputs."

**Response:** We now state explicitly that we did not conduct any empirical studies for evaluating performance, and we recast our contribution as identifying and illustrating situations in which theoretical understanding is likely to help — i.e., generating hypotheses for any systematic studies that would be required to _prove_ the benefit.

 Change — Introduction:

**Original:** "Despite growing interest in interpretability and transparency, there is limited empirical work exploring how theoretical understanding of LLM mechanisms can enhance clinicians' ability to evaluate model outputs. This study addresses that gap."

**Revised:** "Despite growing interest in interpretability and transparency, there is limited work exploring how a theoretical understanding of LLM mechanisms might enhance clinicians' ability to evaluate model outputs. Instead of testing clinicians directly, this study uses a self-referential, hypothesis-generating design to identify and illustrate concrete situations in which such theoretical understanding is likely to be interpretively useful, thereby motivating the empirical, systematic studies that would be needed to demonstrate its benefits."

 Change — Objectives (added sentence) to "… In addition, we delineate concrete examples and situational characteristics in which such theoretical understanding provides interpretive benefit.", as follows: “We do not aim to measure clinician performance or to establish empirically that theoretical knowledge improves output evaluation; rather, we aim to generate and illustrate hypotheses that future user-based studies can test.”

 1.3 Consensus.app does not validate themes.

**Reviewer comment: **"The authors also used Consensus.app as a validation tool. This is another important concern. An AI-assisted literature search platform can help identify relevant papers but it does not itself validate GPT-generated themes."

**Response:** Indeed, consensus.app was used to retrieve relevant peer-reviewed literature and to replace potentially hallucinated citations with verifiable ones — not to confirm the model's themes. We have now made this explicit in the Methods and have removed "validation" language (see also 1.5).

 Change — Methods, Processing:

**Original:** "To validate the output and literature references, we performed a literature search using Consensus.app, incorporating the GPT-o1–generated key topics into the search prompts. Two studies, one focusing on rehabilitation medicine (Zhang et al.) and another on clinical interview analysis using synthetic data (Wu et al.), were identified as especially illustrative and were included in the subsequent analysis."

**Revised:** "To cross-check the outputs and to replace potentially hallucinated references with verifiable ones, we performed a literature search using Consensus.app, incorporating the GPT-o1–generated key topics into the search prompts. From the retrieved results we selected two studies — one focusing on rehabilitation medicine (Zhang et al.) and another on clinical interview analysis using synthetic data (Wu et al.) — as especially illustrative; this selection was non-systematic and intended to exemplify, rather than to comprehensively or quantitatively test, the key topics."

 1.4 Limited and selective evidence base.

**Reviewer comment:** "Another concern is the limited and selective evidence base. Only a small number of 'especially illustrative' studies are used to support the final themes."

**Response:** We agree and have framed the selection honestly as non-systematic (Methods, above) and strengthened the corresponding statement in the Limitations. This is appropriate for an illustrative “Brief Report” but should not be read as a representative survey.

 Change — Discussion, Limitations:

**Original:** "The examples analyzed were generated and selected using AI-based tools and therefore represent only a limited sample of possible cases."

**Revised:** "The examples analyzed were generated and selected using AI-based tools, critically examined and assessed by human authors, and represent only a small, non-systematic sample of possible cases rather than a representative or exhaustive survey."

 1.5 Soften the language ("validated").

**Reviewer comment:** "There is also a need for the language of the introduction to be softened. Terms such as 'validated' give a signal like strong evidence."

**Response:** Throughout the text, "validated" is replaced by words such as  "cross-checked”,”identified”,”illustrated."

 Change — Abstract, Methods:

**Original:** "Key topics were extracted from its responses, and illustrative cases were validated using Consensus.app, an AI-based web-search tool."

**Revised: **"Key topics were extracted from its responses, and illustrative cases were identified and cross-checked against the peer-reviewed literature using Consensus.app, an AI-assisted literature-search tool."

 Change — Introduction:

**Original:** "Key topics were extracted from its responses and validated using Consensus.app, an AI-assisted literature search platform."

**Revised:** "Key topics were extracted from its responses and cross-checked against the peer-reviewed literature using Consensus.app, an AI-assisted literature search platform; importantly, this step served to locate corroborating or illustrative studies, not to confirm the validity of the model-generated topics."

 New references added

 Goh E, Gallo R, Hom J, et al. Large Language Model Influence on Diagnostic Reasoning: A Randomized Clinical Trial. JAMA Netw Open. 2024;7(10):e2440969-e2440969. doi:10.1001/jamanetworkopen.2024.40969

 McDuff D, Schaekermann M, Tu T, et al. Towards accurate differential diagnosis with large language models. Nature.2025;642(8067):451-457. doi:10.1038/s41586-025-08869-4

 Lindsey J, Gurnee W, Ameisen E, et al. On the Biology of a Large Language Model. Transformer Circuits Thread. 2025. Available from: https://transformer-circuits.pub/2025/attribution-graphs/biology.html

 Webster CS. Natural and artificial intelligence – the psychotechnical agenda of the 21st century. Journal of Psychology and AI. 2025;1(1). doi:10.1080/29974100.2025.2491445.

 Dohmatob E, Feng Y, Subramonian A, Kempe J. Strong model collapse. In: International Conference on Learning Representations; 2025:15656-15691. Available from: https://proceedings.iclr.cc/paper_files/paper/2025/file/284afdc2309f9667d2d4fb9290235b0c-Paper-Conference.pdf

 We believe these revisions address the reviewers' concerns while preserving the paper's purpose as an exploratory, hypothesis-generating Brief Report. We are grateful for feedback that has materially improved the clarity and the epistemic calibration of the manuscript.
